# Clinical profiling of skin microbiome and metabolome during re-epithelialization

**DOI:** 10.1038/s41598-025-07547-9

**Published:** 2025-07-01

**Authors:** P. Bianchi, C. Jacques, J. Theunis, E. L. Jamin, C. Orlandi, L. Cauhape, S. Alvarez-Georges, A. Alves, A. Simcic-Mori, C. Lauze, E. Gravier, F. Carballido, V. Ribet, S. Bessou-Touya, H. Duplan

**Affiliations:** 1 Pierre Fabre Dermo-Cosmetics & Personal Care, Centre R&D Pierre Fabre, Toulouse, 31025 France; 2 Medical Department, Laboratoires Dermatologiques A-Derma, Les Cauquillous, France; 3 UMR1331 Toxalim (Research Centre in Food Toxicology), Toulouse University, INRAE, ENVT, INP-Purpan, UPS, Toulouse, 31027 France; 4 MetaboHUB-Metatoul, National Infrastructure of Metabolomics and Fluxomics, Metatoul-AXIOM, Toulouse, 31077 France

**Keywords:** Skin microbiome, Skin metabolome, Clinical study, Wound healing, Rhealba oat concentrate, Sodium hyaluronate, Formula, Gene expression analysis, Metabolomics, Predictive markers, Cell biology

## Abstract

We investigated changes in skin microbiome and metabolome linked to wound healing and how these are affected by a formula known to improve re-epithelialization. In a clinical study with 21 subjects, forearm lesions were induced by epidermal laser ablation. The areas were left untreated or treated with the formula. Re-epithelialization was monitored for 18 days. Skin swabs were analyzed for microbiome diversity using 16 S rRNA gene sequence analysis. Selected species analyzed using digital droplet polymerase chain reaction. Metabolomic profiles were analyzed by ultra-high performance liquid chromatography-high-resolution mass spectrometry. Microbiota alpha-diversity (richness and evenness) was markedly reduced by laser ablation and returned to pre-ablation levels on Day 5. Formula application accelerated the re-epithelialization time (RT), which was more efficient for slow healing (RTs of 15–19 days) than quick healing (10–12 day RTs) subjects. The repairing effect was associated with greater microbiota diversity and species-specific growth of commensal bacteria. Levels of several metabolites on untreated skin at the RT and the extent of the impact of the formula were different in slow and quick healers. The formula significantly modified the skin metabolome, whereby metabolites involved in promoting wound healing were increased and metabolites consumed by the commensal bacteria were decreased.

**Supplementary Information:**

The online version contains supplementary material available at 10.1038/s41598-025-07547-9.

## Introduction

Wound healing is a major global health issue. There is high prevalence of chronic wounds^1^, which creates an economic burden due to health care costs, as well as impacting the quality of life^2,3^. Although generally considered to be less problematic, the treatment of acute, minor wounds e.g., cuts and abrasions, should not be overlooked since they are more prevalent than chronic wounds and do not necessarily heal without additional treatment^4^. Normal healing of acute wounds involves a sequence of processes, including inflammation, proliferation and repair, and remodeling^5^. However, if these phases fail to occur and the transition between resolution of inflammation and re-epithelialization is dysregulated, acute wounds can develop into chronic wounds or hypertrophic scars^6,7^.

Standard wound treatment involves moisture balance and infection prevention; however, there have been recent advances in diverse strategies to manage wound healing, leading to improved clinical outcomes^3^. One of these has been possible due to the insights gained from investigating the skin microbiome in patients with chronic wounds. The presence of bacteria during wound healing can worsen the injury; therefore, these pathogens should be removed to be able to heal properly^8^. However, it has been suggested that repopulation of the wound with resident commensal organisms accelerates wound healing and prevents wound infection by opportunist pathogens^9^. Commensal microbiota, and more specifically, *Staphylococcus epidermidis* (*S. epidermidis*, the most prevalent commensal microorganism), are beneficial through indirect competition^10^, occupying the skin and metabolizing aromatic amino acids present on the skin to trace amines, which accelerate wound healing^11^. The diversity of the resident commensal microbes prevents domination by a single pathogen^12–14^. Thus, balance between commensal and pathogenic microbiota is very important in the wound repair process. Several studies have shown that modulating wound microbes can improve wound healing; however, most of them have been conducted in mice^11,15–18^ or in ex vivo human skin^10^. In one of the few in vivo studies in humans, topical application of a broad-spectrum antibiotic, Neosporin, to punch biopsy lesions in 6 volunteers delayed wound closure^15^. This was linked to the decreased abundance of all microbes, even the pathogenic bacterium, *Staphylococcus aureus* (*S. aureus*), and an alteration of the inflammatory pathways in the skin which promote wound healing.

Human skin is a complex ecosystem where microbiota and skin cells interact with each other via multiple molecular and functional interactions. These interactions can be monitored by measuring associated metabolites^19^. The metabolome is composed of all the low molecular weight compounds of endogenous nature, which are important substrates, by-products, and building blocks of many different biological processes, but also of exogenous compounds and their biotransformation by the body. Metabolomics is now a well-established high-throughput systems biology omics technology capable of generating high-resolution and high-quality data on the physiological and metabolic state of a target tissue. While there have been a handful of studies measuring the metabolome of human skin of patients with chronic leg ulcers at a single timepoint^20–23^, there are very few clinical studies reporting results from metabolomics and microbial diversity analysis measurements before and after acute skin jury, as well as during re-epithelialization within the same experiment. Therefore, we conducted a clinical study in which lesions were induced on the forearms of healthy subjects by epidermal laser ablation, after which re-epithelialization was monitored for 19 days. Skin swabs taken before and at various times after ablation were analyzed for microbiota and metabolomic profiles. In addition, we evaluated how the microbiome diversity and metabolome interactions were modulated by a formula known to improve wound-healing. This formula contains Rhealba oat concentrate^24–26^ and sodium hyaluronate^27^, both of which are known to accelerate wound closure. This study provides further insight into the links between changes in the skin microbiome with changes in the metabolome during wound healing and identifies key pathways involved in this process.

## Results

### Re-epithelialization time (RT)

**Fig. 1:**
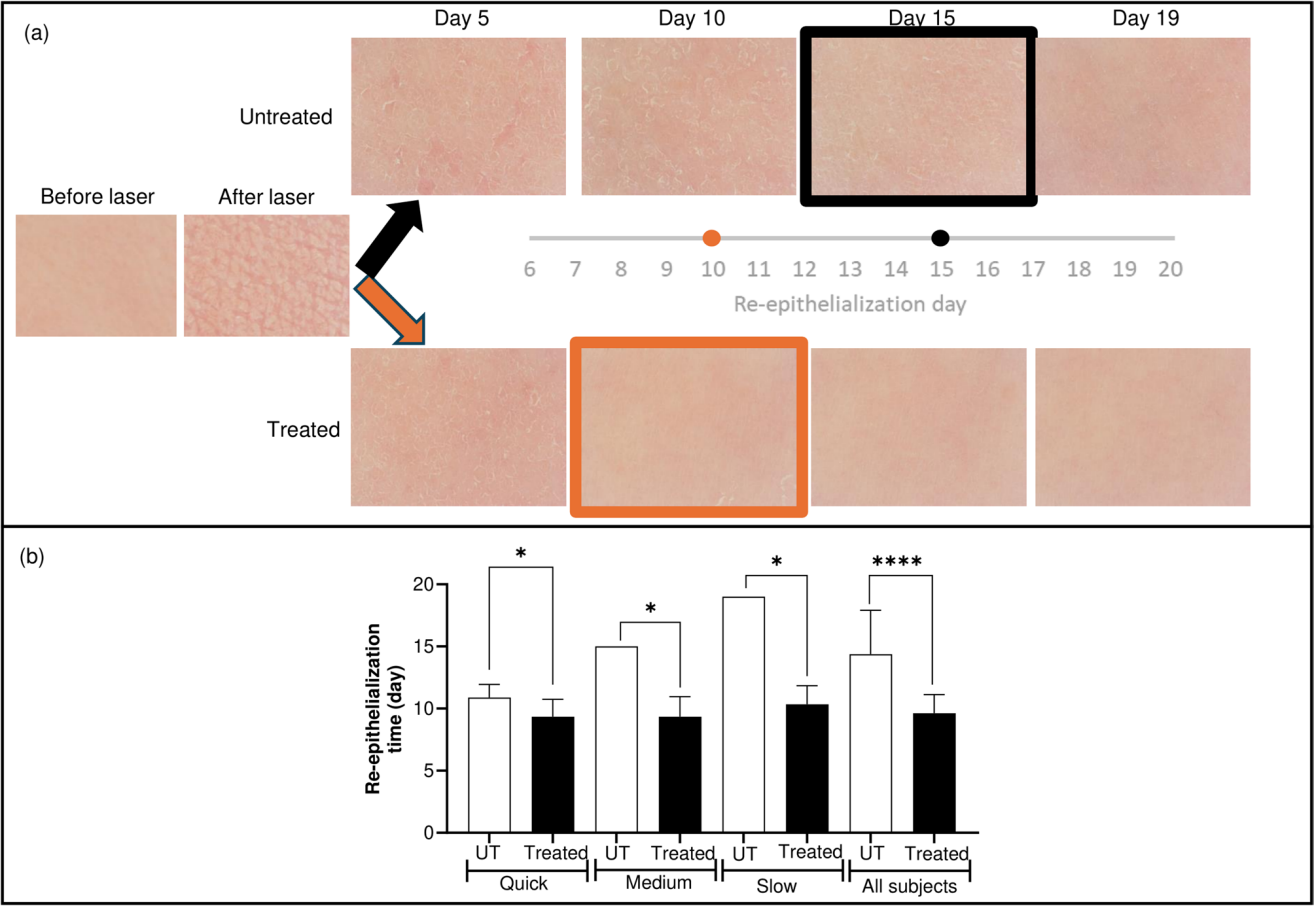
(**a**) Images of skin showing the day on which re-epithelialization was complete. Images were captured using C-Cube imaging and are from a subject reflecting the median result. (**b**) Re-epithelialization time after epidermal ablation in subjects with quick (10–12 days), medium (15 days) and slow (19 days) re-epithelialization times (RT). Values are expressed as a mean number of days + SD for formula treated skin (“Treated”, black bars) versus untreated skin (UT, white bars). Statistically significant differences between treated versus untreated values, using a generalized linear mixed model, are denoted by * (where *p* < 0.05) or **** (where *p* < 0.001).

Figure 1a shows representative images of the skin areas before and directly after epidermal ablation, as well as over the subsequent days of re-epithelialization (up to Day 19) with and without application of the formula. After epidermal ablation, the mean time for untreated skin of all 21 subjects to re-epithelialize was 14.4 ± 3.5 days (ranging from 10 to 19 days) (Fig. 1b). The RT was reduced by 33% to 9.6 ± 1.5 days (ranging between 8 and 12 days) by applying the formula after epidermal ablation. Since the RT in untreated skin varied across subjects (see Supplementary Fig. 1), the RT results were separated into categories of “quick healers” (10–12 days, 9 subjects), “medium healers” (15 days, 6 subjects) and “slow healers” (19 days, 6 subjects) (Fig. 1b). This showed that the effect of treatment with the formula was more extensive for slow healer subjects with long RTs (a reduction of 8.7 days) than quick healer subjects with a short RT (a reduction of 1.1 day).

### Changes in microbiome

**Fig. 2:**
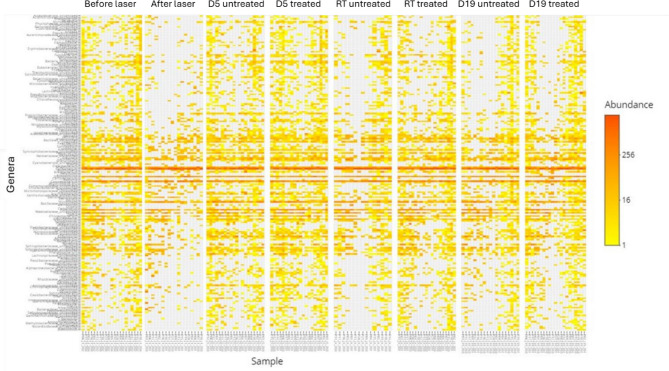
Heatmap of the abundance of bacterial genera (annotated in the y-axis) for each subject (annotated in the x-axis) before and after laser ablation and during the re-epithelialization process. RT (re-epithelialization time) is the day on which re-epithelialization was complete.

**Fig. 3:**
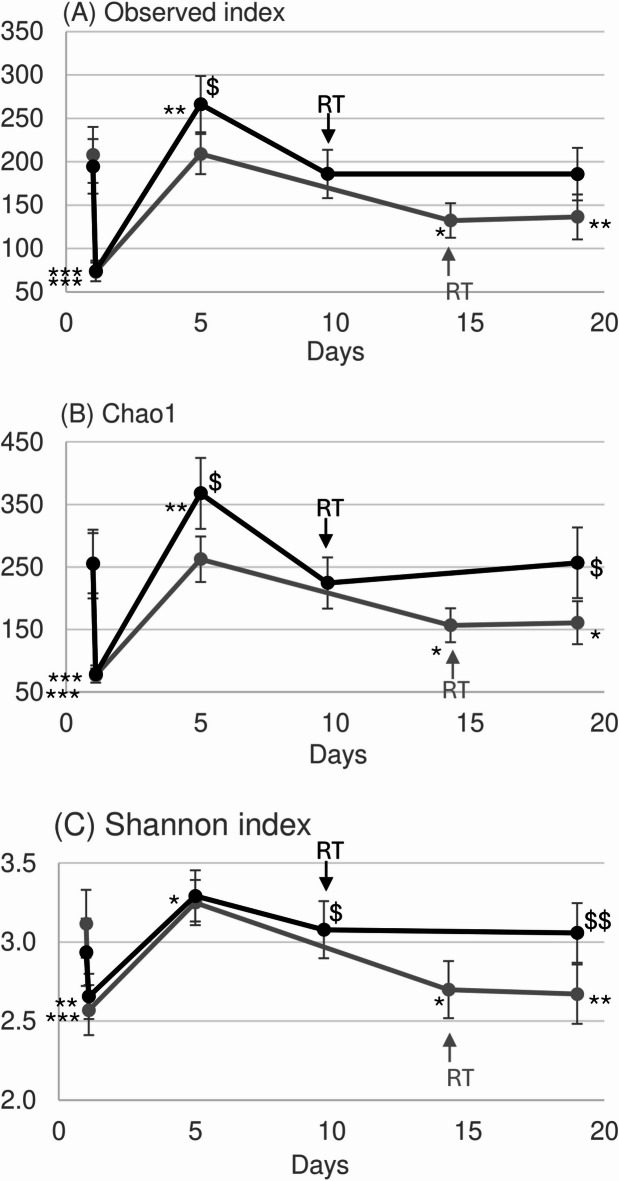
Diversity of taxa based on indices representative of the richness: The Observed index (**a**) and Chao1 (**b**) and evenness: Shannon index (**c**) from samples from untreated (gray symbols and lines) and formula treated (black symbols and lines) skin. The Observed index is the actual number of taxa (count above 0) for each sample; (**b**) Chao1 is an estimated number of taxa not observed (low sequencing depth, bioinformatics filtering etc.) and the Shannon index is the alpha diversity of a bacterial sample (distribution of the abundance). Values are a mean ± SD. RT = re-epithelialization time. Statistically significant differences between the value before laser treatment (first data point) and after treatment (black curve) are denoted by $ (where *p* < 0.05) or $$ (where *p* < 0.01). Statistically significant differences between values after treatment (black curve) versus untreated (grey curve), using a generalized linear mixed model, are denoted by * (where *p* < 0.05), ** (where *p* < 0.01) or *** (where *p* < 0.001).

A first exploration of the sequencing data is represented in the heatmap of the 200 most abundant bacterial genera (Fig. 2) for all subjects before and after epidermal ablation and during the re-epithelialization process. The heatmap clearly shows that epidermal ablation eradicated a substantial number of genera (visualized as the large proportion of grey in the figure) and how the abundance of the genera evolved throughout the healing process. Moreover, it also clearly indicates the positive impact of the formula in maintaining the microbiota. These observations were confirmed by the diversity indices, which were all statistically significantly lower than before epidermal ablation, i.e., a decrease in richness (Observed index and Chao1) and evenness (Shannon index) (Fig. 3). On Day 5 after epidermal ablation, none of the alpha-diversity indices measured for untreated skin were significantly different from values measured just before epidermal ablation. By contrast, all the diversity indices for skin treated with formula were statistically significantly higher than before epidermal ablation, indicating an increase of the richness (*p* < 0.005) and a small increase of the evenness (*p* < 0.05). In addition, the change of bacterial richness (Observed index and Chao1) between Day 5 and the pre-ablation day was statistically significantly (*p* < 0.05) higher in formula treated skin compared with untreated skin. At the RT (Day 10, denoted by RT in Fig. 3), the bacterial profile of formula treated skin was not statistically significantly different from the initial levels before epidermal ablation. By contrast, there was a significant loss of richness (Observed index and Chao-1) and evenness (Shannon index) in the skin of the untreated subjects at the RT (Day 15, denoted by RT in Fig. 3). At the last measurement time (Day 19), the bacterial profile of the treated skin was still similar to the initial profile before epidermal ablation. However, the evenness and richness of the bacterial profile of untreated skin remained lower than the initial value (Fig. 3). In addition, the change of bacterial richness (Chao1) and evenness (Shannon index) between Day 19 and Day 1 (before ablation) were statistically significantly (*p* < 0.05) higher in formula treated skin compared with untreated skin. The same results for fungal diversity were obtained using ITS1 gene sequencing analysis (data not shown).

**Fig. 4:**
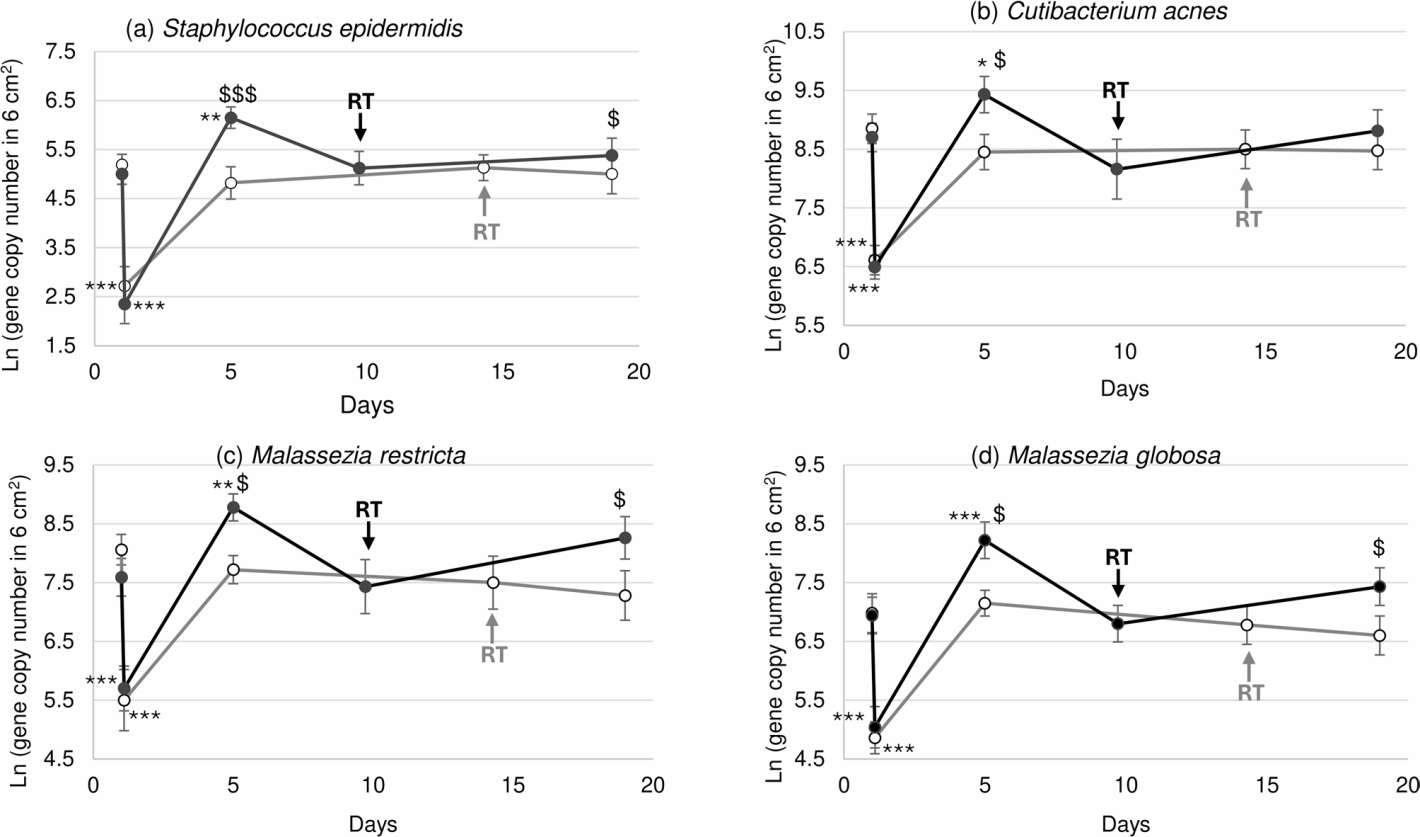
Commensal bacteria and yeast quantification. Mean (± SEM) (**a**) *S. epidermidis*, (**b**) *C. acnes*, (**c**) *M. restricta* and (**d**) *M. globosa* quantification expressed as ln of gene copy number on the area sampled. The re-epithelialization times (RT) are shown by the arrows. Statistically significant differences between the value before laser treatment (first data point) and after treatment (black curve) are denoted by * (where *p* < 0.05), ** (where *p* < 0.01) or *** (where *p* < 0.001). Statistically significant differences between values after treatment (black curve) versus untreated (grey curve), using a generalized linear mixed model, are denoted by $ (where *p* < 0.05) or $$ (where *p* < 0.01).

In addition to the alpha diversity analysis, we conducted a specific analysis on several bacterial (*S. epidermidis*, *Cutibacterium acnes* (*C. acnes*)) and fungal (*Malassezia restricta* (*M. restricta*) and *Malassezia globosa* (*M. globosa*)) species measured at different times after epidermal ablation. This indicated a trend in changes which were comparable to the heatmap results (Fig. 4). The abundance of all four species decreased sharply just after laser treatment, indicating that removal of the epidermis also removed the microbiota. As observed with the diversity indices, 5 days after epidermal ablation, the four taxa returned to baseline levels on untreated skin but were significantly higher in samples from skin treated with formula. At the RT, the abundance of all four taxa were comparable with their initial levels. At the end of the study (Day 19) the abundance of all four taxa on untreated skin continued to decrease compared to treated skin.

### Metabolomic analysis

**Fig. 5:**
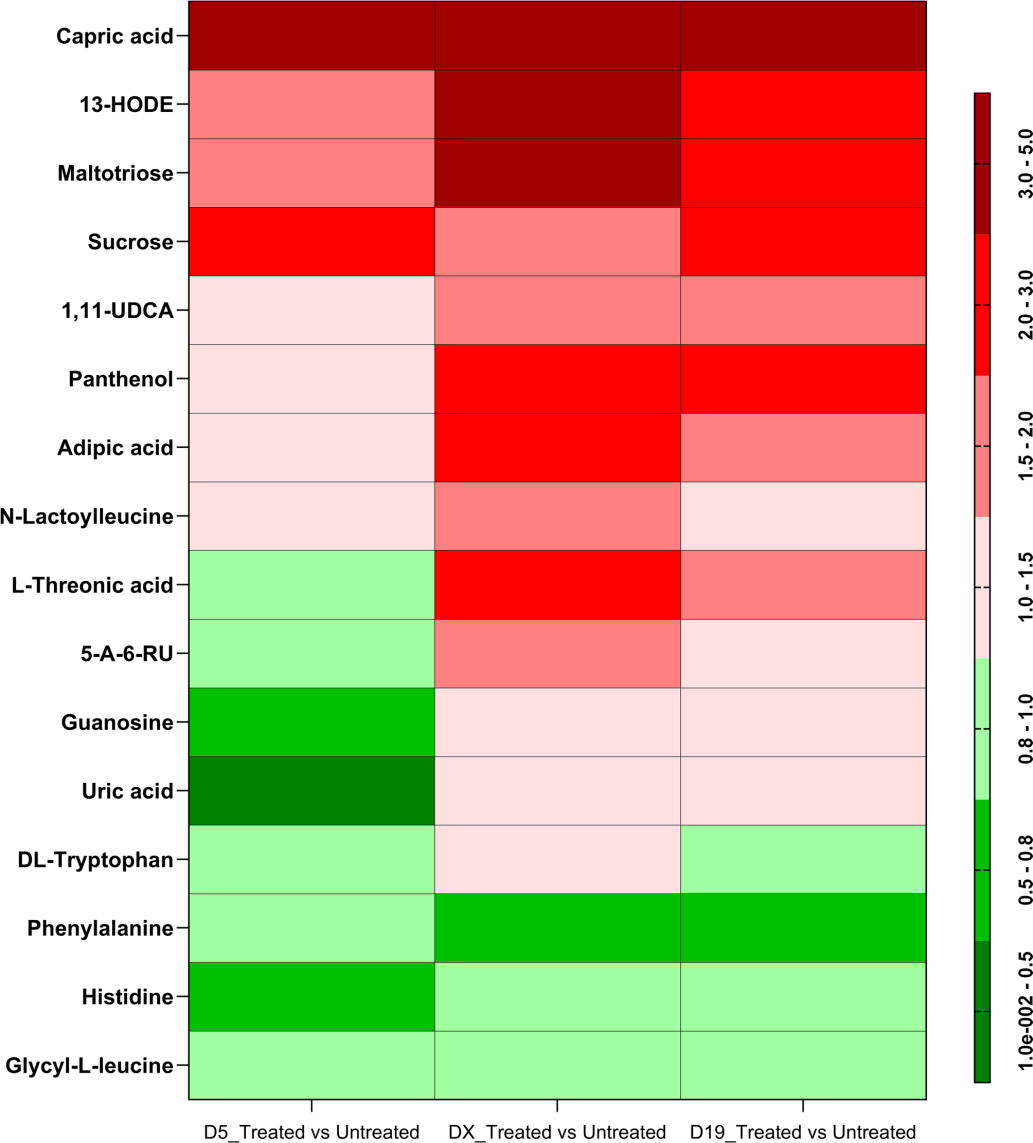
Heatmap of skin swab metabolites significantly (Wilcoxon signed-rank test) deregulated after epidermal ablation with and without treatment with the formula. Fold change upregulation is shown in red and downregulation in green. D5 = Day 5 after epidermal ablation; DX = re-epithelialization time specific to each subject; D19 = Day 19 after epidermal ablation.

**Fig. 6:**
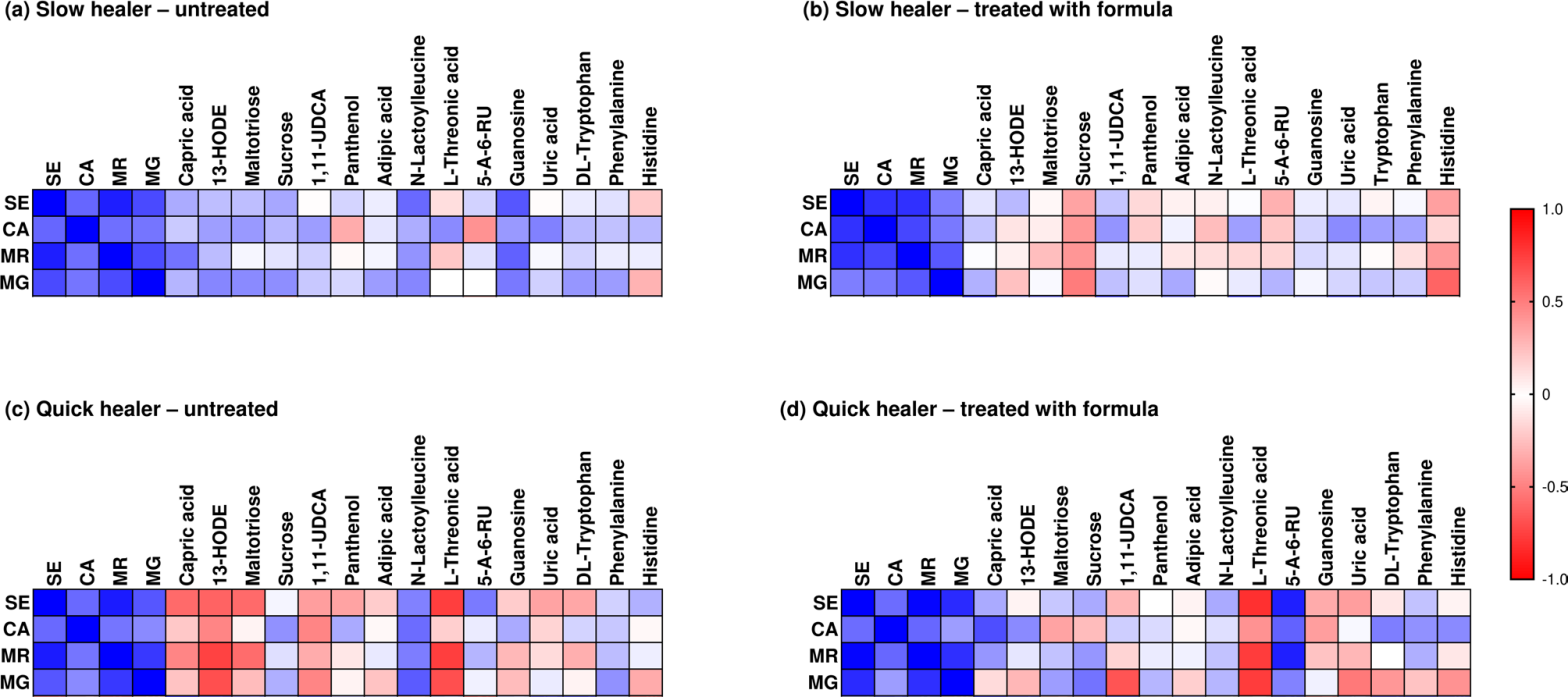
Heatmap showing the Spearman’s rank correlation analysis between identified metabolites and bacterial (*S. epidermidis* (SE), *C. acnes* (CA)) and fungal (*M. restricta* (MR) and *M. globosa* (MG)) species in untreated (**a**, **c**) and formula treated (**b**,**d**) slow (**a**,**b**) and quick (**c**,**d**) healers (medium healers were excluded from this analysis). The cells are colored based on the correlation coefficient between the significantly changed bacteria and metabolites. Blue denotes a significant positive correlation (*P* < 0.05), red denotes a significantly negative correlation (*P* < 0.05), and white denotes that the correlation was not significant (*P* > 0.05) Blue indicates a positive correlation and red indicates a negative correlation – the scale of which is shown in the scale bar.

Sixteen metabolites, identified at levels 1 and 2 according to the metabolomic standard initiative nomenclature, were statistically significantly upregulated by formula compared to untreated ablated skin (visualized in a heatmap in Fig. 5). The correlations between the metabolite and the abundance of the bacterial (*S. epidermidis*,* C. acnes*) and fungal (*M. restricta* and *M. globosa*) species in slow and quick healers are visualized in heatmaps in (Fig. 6). The main classes of metabolites that were statistically significantly modulated by the formula were vitamins, lipids, sugars, purine, and amino acids. Most of these were generally up-regulated, with the exception of amino acids, including phenylalanine, histidine and glycyl-L-leucine, which were all downregulated at each time point by formula compared to untreated samples. Guanosine and the purine breakdown product, uric acid, were also downregulated but only on Day 5.

**Fig. 7:**
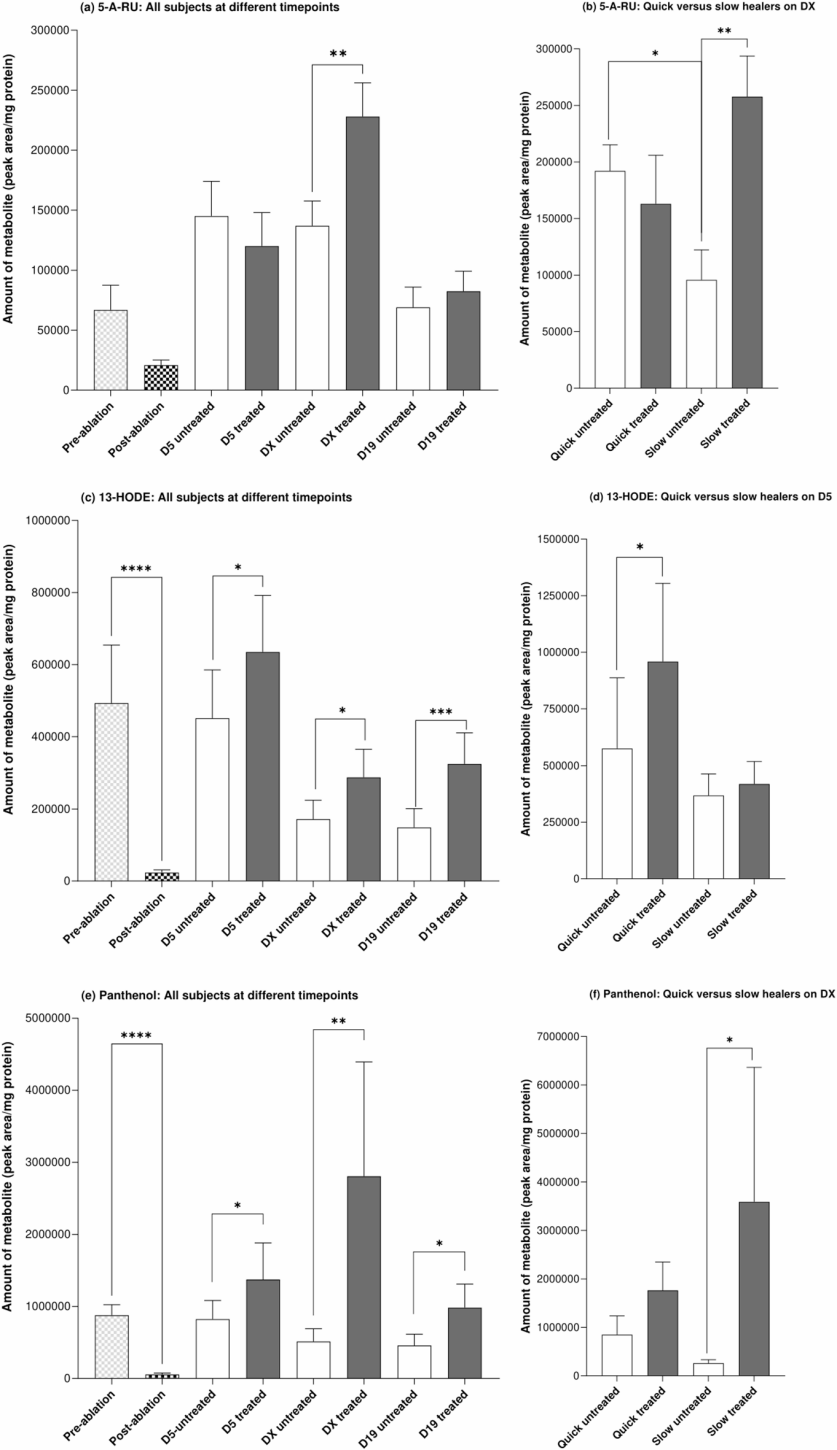
Levels of 5-A-RU (**a**,**b**), 13-HODE (**c**,**d**) and panthenol (**e**,**f**) before and after epidermal ablation and the effect of the formula on their levels at different times of re-epithelialization (DX). Comparison of the levels of 5-A-RU (**b**), 13-HODE (**d**) and panthenol (**e**) in swabs from quick (re-epithelization at Day 10 or 12) and slow healer (re-epithelization at Day 15 or 19) subjects (medium healers were excluded from this analysis). Values are metabolite peak area/mg protein and are a mean + SEM. Statistical significances between values, Wilcoxon signed-rank test, are denoted by * (where *p* < 0.05), ** (where *p* < 0.01), *** (where *p* < 0.001) and **** (where *p* < 0.0001).

One metabolite known to be linked to bacterial metabolism is 5-amino-6-(D-ribitylamino)uracil (5-A-RU). This was supported by the results of this study in which the amount of 5-A-RU in untreated skin samples was positively correlated with the abundance of *S. Epidermidis* and *C. acnes* but not the fungal species (Fig. 6a and c). 5-A-RU was decreased by epidermal ablation (although this did not reach statistical significance) but statistically significantly up-regulated by the formula compared to untreated skin at the time of re-epithelialization (denoted as DX in the graphs) (Fig. 7a). To evaluate the correlation of changes of each metabolite with the rate of wound healing, values for subjects were separated into those who were classified as “quick healers” (re-epithelization at Day 10 or 12) and “slow healers” (re-epithelization at Day 15 or 19). This revealed a statistically significant difference (*p* < 0.05) in the amount of 5-A-RU in samples from untreated quick and slow healers at the time of re-epithelialization (the amount was 2.0-fold lower in slow healers) (Fig. 7b). There was no significant difference in the levels of 5-A-RU with and without formula treatment in samples from quick healers. By contrast, the amount of 5-A-RU in formula treated skin samples was markedly and statistically significantly (*p* < 0.01) increased (by 2.7-fold) in samples taken from slow healers. The positive correlation between the abundance of *S. epidermidis* and *C. acnes* and 5-A-RU was increased further in quick healers treated with formula (Fig. 6d). By contrast, this correlation for slow healers changed from a weak positive correlation in untreated samples (Fig. 6a) to a weak negative correlation in treated samples (Fig. 6b).

**Fig. 8:**
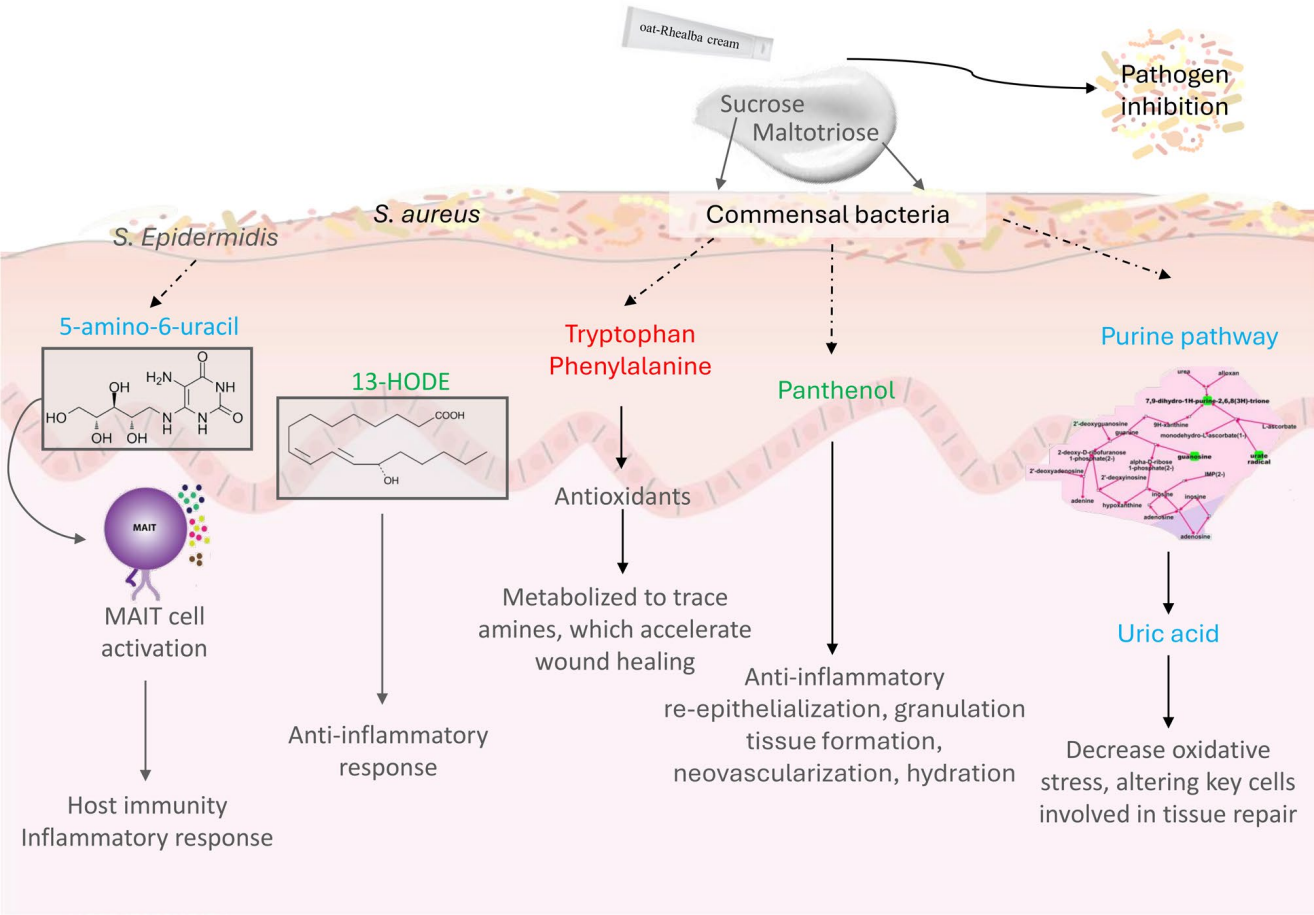
Overview of the effects of the formula on the microbiome and metabolites during the wound healing process. Metabolites in red were down-regulated by the formula, metabolites in green were up-regulated by the formula and metabolites in blue were down-regulated on Day 5 and then up-regulated by the formula.

Maltotriose was the most upregulated sugar, especially at the reepithelization time, but other sugars such as sucrose and threonic acid (a metabolite of threose) were also upregulated in formula treated compared to untreated samples (Fig. 5). Notably, there was a difference between slow and fast healers with respect to the correlations of these sugars with bacterial and fungal species (Fig. 6). For example, there was a strong negative correlation between maltriose and both bacterial species in untreated quick healers (Fig. 6c) but not slow healers (Fig. 6a). This negative correlation in quick healers changed to a weak positive correlation by treatment with the formula (Fig. 6d).

A notable metabolite modified by formula was the lipid, 13(S)-hydroxy-9Z,11E-octadecadienoic acid (13-HODE), which is involved in anti-inflammatory pathways. This was statistically significantly decreased by 21.1-fold as a result of epidermal ablation (Fig. 7c). By Day 5, the levels in untreated skin were comparable with pre-ablation values but subsequently decreased by 2.9- and 3.1-fold on the re-epithelialization day and on Day 19 compared to pre-ablation levels, respectively. The application of formula resulted in statistically significantly higher levels of 13-HODE recovered in skin samples at each timepoint compared to samples from subjects without treatment (1.4-, 1,7- and 2.2-fold higher at Day 5, DX and Day 19, respectively). Unlike 5-A-RU, the levels of 13-HODE in untreated samples were similar in slow and quick healers at all timepoints measured; however, the effect of formula was linked to the rate of healing, whereby there was a statistically significant increase of 1.7-fold by formula in quick healers but not slow healers on Day 5 (Fig. 7d). Interestingly, 13-HODE was slightly positively correlated to all bacterial and fungal species in untreated slow healers and strongly negatively correlated with all four species in samples from untreated quick healers (Fig. 6a and c). In formula-treated samples from slow healers, there was a positive correlation between 13-HODE and *S. epidermidis* only (Fig. 6b), whereas there was a positive correlation between 13-HODE and *C. acnes* in quick healers (Fig. 6d).

Panthenol, also known as provitamin B_5_, was statistically significantly decreased by 15.6-fold as a result of epidermal ablation (Fig. 7e). This was statistically significantly upregulated by formula compared to untreated skin at each time point. Notably, the amount of panthenol in formula treated samples was higher than pre-ablation levels, especially at the re-epithelialization day (3.2-fold higher). The amount of this metabolite in untreated skin was marginally (but not statistically significantly) lower in slow healers than quick healers; moreover, the increase in this metabolite due to treatment with formula was more extensive in slow healers (13.8-fold increase) than in quick healers (2.1-fold increase) (Fig. 7f). The correlation between this metabolite and bacterial and fungal species indicated a difference between slow and quick healers. There was a negative correlation between panthenol and *C. acnes* in untreated samples from slow healers but a positive correlation between these in quick healers (Fig. 6a and c).

## Discussion

The aim of this study was to investigate changes in the skin microbiome and the metabolic pathways involved in wound healing and how these are affected by a formulation designed to improve re-epithelialization.

### Changes in the microbiome

As it has been suggested that microbiota could improve wound healing^28^, we investigated changes in the microbiome due to epidermal ablation, as well during re-epithelialization with and without treatment with formula. As expected, epidermal ablation resulted in a substantial loss in a wide range of genera, which can be expected since they reside on the outer epidermis and thus removing the epidermis also removes the inhabiting genera. The genera re-established to pre-ablation levels within 5 days regardless of whether the ablated skin was treated with formula or not. However, the richness and evenness of taxa in samples from formula treated (but not untreated) skin were significantly higher than pre-ablation values. This indicates that formula increases the diversity of the resident commensal microbes and prevents domination by a single pathogen. A notable difference in the microbiome between untreated and formula skin samples was evident at the RT and on Day 19, at which time the richness and evenness were maintained in formula treated skin but declined in untreated skin. This indicates that the beneficial effect of formula on wound healing, measured as a significant reduction in the RT, could be linked to modulating the skin microbiome.

*Candida albicans* and *S. aureus* are known to be present in infected wounds and were detected in samples from 2 to 3 subjects. Neither species increased over the wound healing process, with or without treatment with formula (data not shown), indicating that the formulation does not promote their growth. This observation suggests that formula preferentially promotes the growth of beneficial microbiota for wound healing rather than having a non-specific effect on all microorganisms. Yu et al. showed that hypertrophic scars are linked to a microbiome dysbiosis dominated by *S. aureus*^29^. By contrast, *C. acnes* was much more prevalent in healthy skin than in hypertrophic scars. The role of *C. acnes* in wound healing is currently unknown; however, since formula increased its growth as well as improving re-epithelialization, and *C. acnes* is associated with healthy skin, the presence of *C. acnes* seems to reflect a healthy wound healing process. The formula increased the growth of *M. restricta* and *M. globosa*, suggesting potential beneficial effects of these fungal species (at least they are not pathogenic); however, no data are currently available in the literature regarding their role in wound healing.

### Changes in the metabolome

The metabolomic analysis indicated that the main classes of metabolites that were statistically significantly modulated by formula were vitamins, lipids, sugars, purine, and amino acids. Of note, adipic acid does not occur naturally and is thus most likely to have been present in the formula formulation (and in other products used by the subjects since it was present in samples before epidermal ablation). It is used as a skin-conditioning agent and solvent in cosmetic formulations^30^ and is therefore not specifically linked to improving wound healing.

#### Vitamin and vitamin intermediates

The results obtained for *S. epidermidis* are particularly interesting because it has been shown in mice, that commensal microorganisms, and more specifically *S. epidermidis*, produce a metabolite called 5-(2-oxopropylideneamino)-6-d-ribitylaminouracil (5-OP-RU) during vitamin B_2_ synthesis^31,32^. This metabolite is an activator of MAIT (mucosal-Associated Invariant T) cells which are necessary for the wound healing process^32^. Studies in mice showed that animals lacking MAIT cells exhibited a huge delay in wound closure and the addition of 5-OP-RU or *S. epidermidis* were sufficient to improve wound closure^33^. MAIT cells are an abundant class of innate T cells which mediate host immunity to microbial infection by recognizing metabolite antigens derived from microbial riboflavin synthesis presented by the major histocompatibility complex class I (MHC-I)-related protein 1 (MR1)^31^. MAIT cells are also thought to play a role in innate antibacterial immunity through the production of cytokines and direct bacterial killing^33,34^. MAIT cells have both effector function and proliferative capacity^35^. In early life, MAIT cells are primed by commensal bacteria and become tolerant to these species. 5-A-RU is an early intermediate in bacterial riboflavin synthesis and is reported to boost MAIT cell production in mice and improve wound healing^36^. 5-A-RU undergoes a condensation reaction with the reactive carbonyl species, glyoxal and methylglyoxal, to produce the potent MAIT cell antigens, 5-OP-RU and 5-(2-oxoethylideneamino)-6-D-ribitylaminouracil (5-OE-RU)^37^. These MAIT cell antigens are presented via MR1 for recognition by the MAIT cell T cell receptor^31^. In the current study, there was a significant decrease of 5-A-RU due to epidermal ablation. Subsequently, during wound healing and after application of formula, there was an upregulation of this metabolite, with significant differences compared to the untreated area at the time of re-epithelialization. To our knowledge, this is the first time this metabolite has been quantified in human skin. Interestingly, levels of 5-A-RU at the RTs were lower in samples from untreated skin from slow healers than from quick healers, suggesting this metabolite and the abundance of *S. epidermidis* could be linked to faster healing. Moreover, while there was no significant difference observed in levels of 5-A-RU in samples at the RT from untreated and treated skin in quick healer subjects, there was a significant increase in 5-A-RU as a result of formula treatment in slow healers. This suggests that formula increases the growth of *S. epidermidis* thereby increasing re-epithelialization by triggering the MAIT-mediated wound healing.

An important observation in this study was the marked up-regulation of panthenol, also known as provitamin B_5_, by the formula. Panthenol is metabolized to pantothenic acid, which is a vital component of coenzyme A^38^. At high millimolar concentrations, pantothenol inhibits coenzyme A production and thus the growth of *S. aureus*, *S. epidermidis*, and *S. saprophyticus* by inhibiting via type II pantothenate kinase II enzymes present exclusively in *Staphylococci* bacteria^39^. In addition to its antibacterial activity, this metabolite plays a direct role in wound healing by supporting the skin’s natural regenerative processes, promoting hydration, reducing inflammation, and aiding in the wound closure^40^. In wound healing, panthenol helps to improve the skin’s barrier function and promote tissue repair by increasing re-epithelialization, granulation tissue formation and neovascularization^40^. Tissue repair is also increased, and scar formation reduced, by panthenol as a result of the stimulation of proliferation and migration of fibroblasts, which produce collagen, the main structural protein in the skin^41^. The anti-inflammatory effects of panthenol can help reduce swelling and redness around the wound and promote the formation of new tissue and accelerate wound closure^40^. It has moisturizing properties that help to keep the wound bed hydrated, preventing excessive dryness^42^. The increase in panthenol was especially marked at the time of re-epithelialization and on Day 19. As an essential vitamin, the skin cannot produce panthenol naturally, it can only to be obtained from external sources. Only plants and microorganisms, including fungi, can synthesize *de novo* pantothenate. Notably, certain bacteria naturally residing on the skin can produce panthenol from pantothenic acid precursors present in the environment, one of which is *S. epidermidis*^43^. As with other commensal bacteria measured in this study, the highest increase of *S. epidermidis* by formula was on Day 5, which could have led to the increased levels of panthenol observed after this time.

#### Lipids and their metabolites

There was a significant modulation of the lipid mediator, 13-HODE, as well as the metabolite of capric triglyceride, capric acid. Capric triglyceride is present in formula and is likely to have resulted in the formation of capric acid over the course of the healing process. The lipid, 13-HODE was depleted after epidermal ablation and although it recovered to re-ablation levels by Day 5, this was not maintained in untreated healing skin. The application of formula significantly increased levels of 13-HODE in skin samples of all subjects at all timepoints during re-epithelialization. 13-HODE is the major lipo-oxygenation product synthesized in the body from linoleic acid and binds to the nuclear transcription factor, peroxisome proliferator-activated receptor gamma (PPARγ), resulting in anti-inflammatory effects^44^. PPARγ is highly involved in macrophage alternative responses and reprogramming and exerts significant anti-inflammatory actions in various inflammatory and metabolic settings^45^. Recently, PPARs have been shown to be involved in epidermal wound repair during the different phases of the healing process^46^. PPAR seems to be a key mediator of epidermal effects in wound healing by converting the extracellular inflammatory signal into an organized pattern of gene expression leading to survival, migration and differentiation of keratinocytes^46^.

1,11-Undecanedicarboxylic acid was increased by the formula at all timepoints. There is limited information on this long-chain fatty acid but it could act via the same mechanism as 13-HODE i.e., by activating PPARγ. This could accelerate healing by regulating lipid metabolism and glucose homeostasis, as well as increasing cell differentiation and regulating growth^47^.

#### Sugars and their metabolites

Among the sugars, maltotriose was the most upregulated by the formula, especially at the reepithelization time, but other sugars such as sucrose and threonic acid (a metabolite of threose and produced by microbes^48^) were also upregulated. Moreover, sucrose metabolism was a major pathway modulated across the wound healing process. This immediate and extensive supply of energy nutrients is essential to regulate the healing process since all significant wounds lead to a hypermetabolic and catabolic state^49^. Maltotriose belongs to the class of organic compounds known as oligosaccharides and consists of three glucose molecules linked with α-1,4 glycosidic bonds^50^. These complex sugars (including maltose and maltohexose) are major sources of glucose for bacteria^51^. Here, the peak up-regulation of sugars by the formula coincided with the statistically significant increases in microbiome number and richness i.e. RT and on Day 19. These sugar metabolites have a considerable advantage as biomarkers due to their specific uptake by bacteria via the maltodextrin transporter, which is not present in mammalian cells^52^. Sucrose is a nonreducing disaccharide composed of glucose and fructose. It is a selective fermentation initiator that can specifically intensify the fermentation activity of *S. epidermidis* but not *C. acnes*, and has been used to reduce water activity within skin follicles and hence bacterial colonization of wounds^53^. Short chain fructo-oligosaccharides (scFOS) from sucrose promote and sustain the growth of the commensal bacterium, *S. epidermidis*, for up to 24 h^51^. scFOS acts as a prebiotic for *S. epidermidis* but not for *C. acnes* and *S. aureus*, indicating that scFOS selectively enhances the growth of *S. epidermidis*^51^. Since maltotriose and sucrose can be formed from the components of the formula e.g., Xanthan gum and oat extract, application of this formula to skin during wound healing provides nutrients for the growth of commensal bacteria.

#### Purines and metabolites

Various purines such as guanosine and deoxyinosine have been shown to improve wound healing^54,55^. Guanosine was another metabolite for which levels at the RT were lower in samples from slow healers than from quick healers, suggesting this metabolite could be linked to faster healing. The levels of guanosine and its breakdown product, uric acid, were downregulated by the formula on Day 5 after epidermal ablation and then upregulated at the RT and on Day 19. This change in purine and uric acid levels may be linked to the increased healing after application of formula, since derivatives of uric acid can improve healing, likely by reducing oxidative stress^56^.

#### Amino acids

Several aromatic amino acids, namely phenylalanine, tryptophan and histidine, were down-regulated by the formula. These were also generally (if not statistically) lower in quick healers than slow healers, suggesting higher levels of this metabolite during wound healing slows the process down. While the presence of the amino acids themselves is linked with beneficial effects in wound healing (Table 1), it may seem that their down-regulation is detrimental. However, their metabolism by *S. epidermidis* strains to trace amines can be linked to beneficial effects since it is reported that trace amines accelerate wound healing in mice^11^. Glycyl-L-leucine is a branch-chain amino acid. These have also been reported to have beneficial effects in healing wounds of diabetic patients with foot ulcers^57^, and mechanism by which it does so is thought to be associated with anabolic effects on proteins involved in wound healing^49^.

**Table 1:** Summary of the 16 metabolites modified by the formula, together with their roles in wound healing and the consequence of modulation by the formula.

Metabolite	Role in wound healing	Link to microbiome species in untreated skin, impact of formula and associated beneficial effects in wound healing
Vitamin and vitamin intermediates		
5-A-6-RU	An early intermediate in bacterial riboflavin (vitamin B_2_) synthesis (specifically *S. epidermidis*) and activator of MAIT cells which are needed for the wound healing process^32^. The correlation between 5-A-6-RU in quick untreated samples, which became stronger when treated with the cream.	Positively correlated with SE and MR in quick healers. Initially ↓ then ↑ by the formula - increases growth of SE to increase re-epithelialization by triggering the MAIT-cell mediated wound healing.
Panthenol	Provitamin B_5_. Exhibits antioxidant, antibacterial, anti-inflammatory and proangiogenic properties; promotes hydration^40^. Promotes keratinocyte and dermal fibroblast migration and proliferation, resulting in improved re-epithelialization^40^.	Positively correlated with CA in quick healers and negatively correlated with CA in slow healers. ↑ at all timepoints by the formula.
Lipids and lipid metabolites		
Capric acid	None – metabolite of the emollient, capric triglyceride.	Positively correlated with SE, CA, MR and MG in slow healers but negatively correlated in quick healers. ↑ at all timepoints by the formula. Indirect beneficial effects as an emollient.
13-HODE	Anti-inflammatory effects^44^. Activates PPAR mediated epidermal effects in wound healing by converting the extracellular inflammatory signal into an organized pattern of gene expression leading to survival, migration and differentiation of keratinocytes^46^.	Positively correlated with SE, CA, MR and MG in slow healers but negatively correlated in quick healers. ↑ at all timepoints by the formula - increases anti-inflammatory actions.
1,11-UDCA	May act via PPARγ to regulate lipid metabolism and glucose homeostasis along with cell differentiation and growth regulation^47^.	Positively correlated mainly with CA in slow healers but negatively correlated with SE, CA, MR and MG in quick healers. ↑ at all timepoints by the formula. Hypothesis: Increases tissue repair via same mechanism as 13-HODE
Sugars and their metabolites		
Maltotriose	Major sources of glucose for bacteria^51^. Promote and sustain growth of the commensal bacterium, *S. epidermidis*^51^.	Maltotriose and sucrose positively correlated with SE, CA and MG in slow healers. Maltotriose negatively correlated with SE and MR and sucrose positively correlated with CA and MG in quick healers. L-Threonic acid correlated with CA in slow healers and strongly negatively correlated with SE, MR and MG in quick healers. Formula application results in peak up-regulation of all three sugars concomitant with ↑ in microbiome number and richness i.e. RT and on Day 19.
Sucrose		
L-threonic acid	Metabolite of threose. The L-isomer is a metabolite of ascorbic acid (vitamin C). It is reported to be a microbial metabolite^48^.	
Purines and metabolites		
Guanosine	Metabolized to uric acid.	Strong positive correlation with SE, CA, MR and MG in slow healers but negatively correlated with SE, MR and MG in quick healers. Formula application results in initial ↑ and then ↓ – profile mirrors that of its metabolite, uric acid. Improves healing by reducing oxidative stress and altering key cells involved in tissue repair.
Uric acid	Purine metabolite. Antioxidant - an efficient free radical scavenger^56^. Modifies behaviors of vascular endothelial cells, keratinocytes and fibroblasts resulting in accelerating wound healing^56^.	Positively correlated with CA in slow healers but negatively correlated with CA (and SE and MR) in quick healers. Initially ↑ due to its involvement in free radical scavenging, and then ↓. Improves healing by reducing oxidative stress and altering key cells involved in tissue repair.
Amino acids		
DL-tryptophan	Antioxidant^66^.	Generally positively correlated with ≥ 1 species in slow and quick healers. ↓ due to increased abundance of certain bacteria i.e., *S. epidermidis*, which metabolize these aromatic amino acids to trace amines, which accelerate wound healing [11].
Phenylalanine	Antioxidant, supports formation of new blood vessels, accelerates tissue regeneration and aids in collagen synthesis at the wound site^67^.	
Histidine	A deficiency in this amino acid leads to decreased hyaluronan (hyaluronic acid) levels. This plays an important role in tissue repair and in cellular proliferation and migration in skin^68^.	
Glycyl-L-leucine	Branched chain amino acid. Fosters anabolic effects on proteins involved in skin wound healing^49,69^	↓at all timepoints. Hypothesis: Mechanism of beneficial effect of down-regulation is unknown.
N-Lactoylleucine	Derivative of leucine.	Strongly positively correlated with SE, CA, MR and MG in slow and quick healers.
Miscellaneous		
Adipic acid	Skin-conditioning agent and solvent in cosmetic formulations^30^	No strong correlation with SE, CA, MR and MG in slow and quick healers. ↑ at all timepoints. Indirect beneficial effects by skin conditioning.

*1,11-UDCA * 1,11-Undecanedicarboxylic acid, *SE*  *Staphylococcus epidermidis*, *CA*  *Cutibacterium acnes*, *MR* *Malassezia restricta*, *MG*  *Malassezia globosa*, *MAIT* mucosal-associated invariant T, *PPARγ*  peroxisome proliferator-activated receptors gamma.

### Microbiome–metabolome interactions

Correlations were made between the 16 metabolites and specific bacterial and fungal species, which provides an indication of the links between the microbiome and the metabolic profile. Table 1 lists the 16 metabolites which were modified by the formula, their correlation with bacterial and fungal species in untreated skin samples, together with their roles in wound healing and the consequence of modulation by the formula. One metabolite known to be linked to bacterial metabolism is 5-A-RU. This was detected in untreated skin samples and was positively correlated with the abundance of *S. Epidermidis* and *C. acnes* but not the fungal species. The positive correlation was highest in quick healers treated with the formula, suggesting that the presence of these bacterial species is beneficial for healing and enhanced by the formula. Another metabolite which is important for bacteria is maltriose. This is a major source of glucose for bacteria^51^ and is known to promote and sustain the growth of the commensal bacterium, *S. epidermidis*^51^. In the current study, there was a strong negative correlation between maltriose and both bacterial species in untreated quick healers but a positive correlation between maltriose with SE, CA and MG in slow healers. The application of the formula up-regulated this sugar in samples all subjects (together with sucrose and L-threonic acid), which resulted in an increase in microbiome number and richness on the RT and on Day 19. In quick healers, this up-regulation was specifically linked to *C. acnes*. These findings highlight the link not only between the microbiome and metabolic profiles but also between the microbiome and slow *versus* quick healers. Therefore, analyzing the microbiome may help in understanding why some subjects respond differently from others to treatments for re-epithelialization.

### Considerations of the study and future investigations

There are known differences in the microbiome and metabolome between male and females due to factors such as hormonal differences^58^. Therefore, we selected only female subjects to reduce the variability in the endpoint measurements, with the caveat that this only represents changes in females. Future studies could investigate the effect of laser ablation and treatment with the formula on the skin microbiota and metabolome of male subjects.

Given the variability in the RT for the 21 subjects, additional studies could be conducted to increase the number of subjects representing quick, medium and slow healers. However, this initial study provides important insights into the processes involved during acute wound healing, as well as helping to design future clinical studies investigating them.

It should be noted that changes in the microbiome and metabolome of wounds (from injury to healing) as the result of laser ablation (representing superficial damage to epidermis) could be different from those of other commonly encountered wounds such as incisions, lacerations, punctures, and avulsions, which are deeper skin wounds involving the healing of the dermal layer.

## Conclusions

This integrative approach investigating the associations and changes in microbiota and metabolites present on the skin highlights a network of biological interactions that occur during the wound healing process. Microbiota and metabolites linked to inflammation and cell growth were markedly decreased by epidermal ablation, and their subsequent profiles during the healing process were modified by the application of a formulation known to improve wound healing. An overview of the effects of the formula on the microbiome and metabolites during the wound healing process is shown in (Fig. 8). Application of formula to the ablated skin accelerated the RT, which was more profound for slow healers than quick healers. This beneficial effect was associated with greater microbiota diversity and the species-specific growth of taxa present on the skin.

The formula also significantly modified the metabolome of the skin, whereby metabolites involved in promoting wound healing were either increased e.g. panthenol, 5-A-RU and 13-HODE and metabolites metabolized by the commensal bacteria were decreased e.g., phenylalanine. Components of the formula e.g., Xanthan gum and oat extract, can lead to the production of a source of energy for bacteria, such as maltotriose and sucrose. Thus, modifying the sucrose pathway leads to an increase in commensal bacteria, such as *S. epidermidis*, but not pathogenic bacteria such as *Candida albicans* or *S. Aureus*. An interesting finding was that levels of several of the metabolites on untreated skin at the RT were different in slow and quick healers. Likewise, the extent of the impact of the formula on levels of several metabolites was also dependent on whether the subject was a slow and quick healer. Further investigations are needed to analyze the differences in metabolites between slow and quick healers, which may help to identify additional metabolites and pathways important in wound healing.

## Materials and methods

### Test formulation

The tested formula used is a proprietary commercial formulation containing oat Rhealba concentrate and cicahyalumide (a mixture of Rhealba, the dipeptide, L-Ala-L-Glu and hyaluronic acid^59^) and is referred to from hereon as “formula”. This is commercially available and is the property of Pierre Fabre Dermo-Cosmétique. The ingredients are water (Aqua), caprylic/​capric triglyceride, glycerin, Butyrospermum Parkii (Shea) Butter (Butyrospermum Parkii Butter), dimethicone, cetearyl alcohol, glyceryl Stearate, palmitic acid, stearic acid, sodium hyaluronate, Avena Sativa (Oat), (Avena Sativa Leaf/​Stem Extract), alanyl glutamine, Uncaria Tomentosa Extract, batyl alcohol, benzoic acid, caprylyl glycol, cetearyl glucoside, dimethiconol, polyacrylate-13, polyisobutene, polysorbate 20, propylene glycol, sodium hydroxide, sorbitan isostearate, tocopheryl acetate, and Xanthan Gum.

### Subjects

Twenty-one women aged between 22 and 44 years old were included. The inclusion and exclusion criteria are listed in detail in the Supplementary Materials. These included excluding subjects with co-morbidities and/or treatments that could impact microbiota. All had skin Phototype I, II or III, according to the Fitzpatrick classification, and smoked fewer than 10 cigarettes per day (containing nicotine, paper and/or electronic cigarette equivalent). Subjects agreed not to expose their forearms to UV (natural or artificial) light throughout the duration of the study to avoid unwanted skin pigmentation (since the formula did not contain a UV filter) and ensure no further skin damage occurred during the study (which could impact the results).

### Treatment and evaluation visits

Subjects were recruited for 9 visits on Day 1, 2, 3, 5, 8, 10, 12, 15 and 19. On Day 1, a 6 cm² area of both forearms of each subject was epidermally ablated with an erbium YAG laser at Visit 1 (D1). This treatment resulted in the wounded condition. An adjacent area of both forearms was left unwounded, serving as a control to compare how the skin behaves without any damage. Subsequently, wounded and non-wounded areas of one arm of each subject were treated with formula, to assess how the formula affects both wounded skin (to monitor healing process) and normal skin (to monitor potential benefits or side effects). The treatment was applied to only one arm but whether it was the right or left arm was determined randomly.

On Day 2 and Day 3, the arms were assessed by a medical investigator to ensure no severe effects due to the ablation occurred. Samples were not taken on these days to avoid delaying the “normal” re-epithelialization of the wound by using swabs.

In a preliminary study (6 subjects), the microbiota was restored 8 days after laser ablation; therefore, for this study, we analyzed samples prior to and just after the laser ablation and up to 19 days after laser ablation to capture modifications of the microbiota, as well as the re-epithelialization process. Therefore, samples from untreated and treated skin were from four visits: twice on Day 1 (baseline and post-ablation), once on Day 5, once on the day of re-epithelialization (subject-dependent and defined by a medical investigator) and once at the end of the study (Day 19, at which time all subjects exhibited complete re-epithelialization – see Supplementary Fig. 1). A total of 10 samples were obtained from each of the 21 individuals at identical anatomical sites.

Participants were asked to avoid washing for 8 h prior to sample collection. The investigator systematically questioned subjects at each visit to verify compliance with specific conditions. For example, subjects were asked orally at what time the last shower was taken. To ensure consistency in the technique used for sample collection, the same technician collected all samples for the study. This technician is experienced in this practice and was trained specifically for the purpose of collecting samples for DNA and protein quantifications (quality assurance using reference samples was confirmed before conducting this study).

For the sample collection, Copan sterile swabs were premoistened with either saline buffer (NaCl 0.9%, for microbiome (sequencing and ddPCR) analysis) or water (for metabolomic analysis) and used to vigorously rub a 6 cm^2^ area of skin for 40 s. Different solvents were used because saline interfered with metabolomic analysis and water could impair the membrane integrity of some microbiota. Therefore, the most appropriate buffer or medium was used for each method.

### Evaluations

The evaluations included visual assessment of re-epithelialization rates; bacterial sequencing using16S rRNA gene sequence analysis, quantification of selected bacteria and yeast using digital droplet PCR (ddPCR), and untargeted metabolomics analysis using RP-UHPLC-HRMS.

### Visual assessment of re-epithelialization

The re-epithelialization was evaluated by the investigator at the same time of the day for all subjects. The time at which re-epithelialization was complete (the re-epithelialization time (RT)) was evaluated on visits on Day 5, 8, 10, 12, 15 and 19 by a single experienced medical investigator by visual examination and with the assistance of the C-Cube© imaging. The C-Cube Dermoscope Clinical Research Edition camera (Pixience SAS, Toulouse, France) features a high-resolution 10-megapixel CMOS sensor integrated into a specialized probe, coupled with a patented glare-free lighting system for optimal image clarity. It employs True Color^®^ patented technology, ensuring precise rendering of the full spectrum of natural skin tones. The imaging system provides a field of view of 12 × 16 mm with a magnification of ×60, delivering high-quality, standardized images. It enables the analysis of structures as small as 10 microns, offering a detailed examination of skin microstructures. Additionally, it provides accurate color quantification, making it a valuable tool for dermatological analysis and clinical research.

The clinical examination took into account the color of the wound, inflammation signs such as erythema evolution and the presence of crust and oozing.

The reduction in the re-epithelialization time (RT) was calculated according to Eq. (1).

1 $$\:Reduction\:in\:RT\:\left(\%\:untreated\right)=\:\frac{\left({RT}_{UT}-\:{RT}_{Treated}\right)}{{RT}_{UT}}\:\times\:100$$

### Bacterial sequencing – 16S rRNA gene sequence analysis

Library preparation and sequencing were performed by the GeT-PlaGe core facility, INRAe Toulouse. The V1V3 region was amplified from purified genomic DNA with the primers F (5’ CTTTCCCTACACGACGCTCTTCCGATC-AGAGTTTGATCCTGGCTCAG 3’) and R (5’ GGAGTTCAGACGTGTGCTCTTCCGATCT- TTACCGCGGCTGCTGGCAC 3’) using 35 amplification cycles with an annealing temperature of 65 °C. The primers/probes were designed in our laboratory are specific to the species targeted in the study (validated by assessing their specificity to target species). Given the known biases of different V-region primers for 16 S RNAseq, long range 16 S rRNA gene sequencing would have been more accurate; therefore, only the bacterial diversity was compared during the wound healing process. The analysis focused on overall microbial diversity and community structure, without assessing changes at the genus level. Because MiSeq enables paired 300-bp reads, the ends of each read are overlapped and can be stitched together to generate extremely high-quality, full-length reads of the entire region in a single run. Single multiplexing was performed using a homemade 6 bp index, which were added to reverse primer during a second polymerase chain reaction (PCR) with 12 cycles using forward primer (5’ AATGATACGGCGACCACCGAGATCTACACTCTTTCCCTACACGAC 3’) and reverse primer (5’ CAAGCAGAAGACGGCATACGAGAT-index-GTGACTGGAGTTCAGACGTGT 3’). The resulting PCR products were purified using Proteigene Clean NGS beads at a ratio of 0.6X to eliminate primer dimers. The purified amplicons were pooled equimolarly and then the pool was denatured before loading onto the Illumina MiSeq cartridge according to the manufacturer’s instructions. The quality of the run was checked internally using PhiX, and then each pair-end sequence was assigned to its sample with the help of the previously integrated index.

Sequences were processed using Mothur (version 1.44.0), according to the MiSeq SOP pipeline^60^. Barcodes, primers, and sequences showing homopolymers of more than 8 bp were discarded. Sequences showing 100% homology were grouped into unique sequences and then into OTUs (operational taxonomic unit, based on 97% homology). Sequences were then assigned to match a sequence in Greengenes (August 2013 release of gg_13_8_99, containing 202,421 bacterial and archaeal sequences) to identify the genus level. Counts were normalized according to the number of total sequences to reach relative abundances.

### Digital droplet PCR (ddPCR)

Selected bacteria and yeast were quantified by ddPCR (PCR based on water-oil emulsion droplet technology). Briefly, DNA was extracted from each swab using the DNA Investigator kit, according to the manufacturer’s instructions (QiaCube, Qiagen). DNA was detected and quantified using the QX200 Droplet Digital PCR system (Bio-Rad Laboratories Inc., Pleasanton, CA, USA), according to the manufacturer’s instructions. Template DNA (5 µL), 1 x Bio-Rad Residual DNA Quantification Supermix, forward primer, reverse primer, probe (listed in Supplementary Table 1) and UV-radiated water was mixed to a final volume of 20 µL, with an estimated DNA concentration range of 1–120000 copies per 20 µL reaction. The PCR mix was put into a droplet generator cartridge, droplet generator oil was added, and the cartridge was placed into a droplet generator. The generated droplet emulsion was transferred to a 96-well PCR plate and amplified in a thermal cycler. The ddPCR program “2 steps” was used: 10 min at 95 °C, 30 s at 94 °C, followed by 40 cycles: 1 min at 59 °C and 10 min at 98 °C, after which the samples were stored at 12 °C. After amplification the plates were transferred to a droplet reader. The analysis was used to determine the fraction of PCR positive droplets in the original sample, after which the absolute target DNA template concentration was calculated.

### Untargeted metabolomics analysis

Untargeted metabolomics analysis was conducted using ultra-high performance liquid chromatography-high-resolution mass spectrometry systemUHPLC (RP-UHPLC-HRMS). Samples prepared in 150 µL of water: acetonitrile (9:1, v: v) were injected in a UHPLC system ACQUITY from Waters (Manchester, UK), using water: methanol: acetic acid (95:5:0.1, v: v:v) as mobile phase A and methanol: acetic acid (99.9:0.1, v: v) as mobile phase B, at a flow rate of 0.3 mL/min. The following gradient program was used: from 0 to 30 min: 0–100% of B, from 30 min to 34 min: 100% of B. The separation was achieved at 40 °C with a hypersil Gold C18 column (100 × 2.1 mm, 1.9 μm) from Thermo Scientific (Les Ulis, France). The following parameters of electrospray were applied: capillary voltage 0.5 kV, sampling cone voltage 30 V, source temperature 120 °C, desolvation temperature 250 °C, cone gas flow rate 50 L/h and desolvation gas flow rate 600 L/h in the positive mode; capillary voltage 0.5 kV, sampling cone voltage 30 V, source temperature 120 °C, desolvation temperature 250 °C, cone gas flow rate 30 L/h and desolvation gas flow rate 700 L/h in the negative mode. High resolution mass spectra were acquired with a QToF Synapt G2-Si mass spectrometer from Waters (Manchester, UK), between m/z 80 and 800 in the sensitivity and the centroid modes. Samples were analyzed randomly, and an experimental blank sample consisting of an extract of sterile swab, as well as a QC sample consisting of the pool of all samples were analyzed 23 times and distributed throughout the sequence of injections. The high-resolution mass analyzer was calibrated in each ionization mode based on the supplier’s protocol and calibration mixtures. Peak areas for 13(S)-Hydroxyoctadeca-9Z,11E-dienoic acid (13-HODE), 5-amino-6-(D-ribitylamino)uracil (5-A-RU), guanosine and histidine were from the positive mode and all other metabolites listed in the results were from the negative mode.

Raw data were converted in mzML format using Proteowizard and CWT and then processed using XCMS offline software with a centwave algorithm (ppm = 20, peak width = (10,50), snthresh = 3, bw = 5, mzwid = 0.01). All data analysis steps were performed using workflow4metabolomics, a collaborative portal dedicated to metabolomics data processing. Only features displaying a signal ratio QC/Blank above 2 and a relative standard deviation in QC below 30%, were kept for statistical analyses.

Compounds were identified using tandem mass spectrometry (MS/MS) by injection of samples into an Acquity I-Class UPLC system (Waters, Manchester, UK) linked to an LTQ-Orbitrap XL mass spectrometer (Thermo Scientific, Les Ulis, France). Validation of identification was based on the nomenclature of the metabolomics standards initiative^61^ using in-house databases of retention times, with MS spectra and MS/MS spectra for level 1 and HMDB for level 2 identifications.

### Microbial diversity analysis

The alpha diversity of sequencing data was evaluated according to the Observed index, Chao1 and the Shannon index. The Observed index is the actual number of taxa (count above 0) for each sample. Chao1 is the total number of taxa in a community, taking into account both the observed taxa and the unobserved taxa (low sequencing depth, bioinformatics filtering). It assumes that rare taxa are more likely to be missed in sampling, and it uses the abundance distribution of the observed taxa to estimate the number of unobserved taxa. The Observed index and Chao1 are representative of richness (the number of taxa present). The Shannon index measures both richness and evenness (how equally abundant the taxa are). Diversity analyses were performed after rarefaction normalization^62^. A mixed linear model explaining the changes between Day 1 (D1) before epidermal ablation and D1, Day 5 (D5), re-epithelialization time (RT) or Day 19 (D19) after epidermal ablation, was performed for each alpha diversity index The arm (left/right), the group (treated/untreated), the visit and the group*visit interaction were included as fixed effects. The subject was included as a random effect and the parameter value on D1 before epidermal ablation (baseline value) was included as a covariate.

### Statistical analyses

All statistical tests were two-sided and the type I error (α) was set at 5%. Data were analyzed and visualized using R software version 4.2.1^63^.

For 16s and ITS1 analyses, the phyloseq R package version 1.42.0^64^was used. The “rarefy_even_depth”, “plot_heatmap” and “estimate_richness” functions were respectively used for rarefaction normalization, heatmap visualization, and computation of alpha-diversity indices. The lme4 R package version 1.1–33 was used to fit linear mixed-effects models.

For ddPCR analyses, a generalized linear mixed model for repeated measures was used with group, area, visit and interaction group / visit as fixed effect, baseline as covariate and subject as random effect.

For metabolomics analyses, a Wilcoxon signed-rank test was used on the log2 of features’ intensities normalized by protein content. In case of inter-group comparisons, tests were applied on the changes between D1 before epidermal ablation and D1, D5, RT or D19 after epidermal ablation. A multiple testing correction (Benjamini-Hochberg procedure^65^) was applied on the raw p-values. For intra-group analysis, fold- changes were computed as the median of each individual fold change across all pairs.

In order to compare groups on the re-epithelialization time, a generalized linear mixed model was used, with “group” and “area” as fixed factors, and subject as random factor.

## Electronic supplementary material

Below is the link to the electronic supplementary material.

Supplementary Material 1

## Data Availability

The data will be shared via the FDA Clinicaltrials.gov under the trail registration numbers NCT06387277. Further enquiries can be directed to the corresponding author, Pascale Bianchi (pascale.bianchi@pierre-fabre.com).
