# Supplementary material for: Clinical profiling of skin microbiome and metabolome during re-epithelialization
Source: Sci Rep. 2025 Jul 1;15:22282. doi: 10.1038/s41598-025-07547-9 (PMC12219420; doi:10.1038/s41598-025-07547-9)
Supplement: Supplementary file 1 — Supplementary Material 1 [file 41598_2025_7547_MOESM1_ESM.pdf]

Supplementary Materials: Clinical Profiling of Skin Microbiome and Metabolome During Re-epithelialization

P. Bianchi[1*], C. Jacques[1*], J. Theunis[1], E.L. Jamin[3, 4], C. Orlandi[3,4], L. Cauhape[1], S. Alvarez-Georges[1], A. Alves[1], A. Simcic-Mori[1], C. Lauze[1], E. Gravier[1], F. Carballido[2], V. Ribet[1], S. Bessou-Touya[1] and H. Duplan[1]

Materials and methods

Subject inclusion criteria

1. Female, from 20 to 45 years old.
2. Phototype I, II or III according to the Fitzpatrick classification.
3. Subject smoking ≤ 10 cigarettes / day containing nicotine, paper and/or electronic cigarette equivalent.
4. Subject agreeing not to expose his forearms to UV (natural or artificial) throughout the duration of the study and up to 3 months after the end of participation in the study
5. If the subject is a woman of childbearing age:
   i. Subject who used an effective method of contraception, evaluated by the investigator for at least 8 weeks before inclusion in the study
   ii. Subject agreeing to continue using it during all the study and up to 5 days after the last products application, in order to avoid pregnancy during exposure to the study products
6. Absence from participation in a clinical research protocol
7. Signature of the informed consent form.

Exclusion criteria
Subjects meeting at least one of the following criteria are not included:

Population: Male subjects

Diseases or skin condition:

1. Subject with known immunodeficiency
2. Subject with known acquired immunodeficiency syndrome
3. Subject with known infectious hepatitis or a known history of hepatitis B or C
4. Subject with a known history of allergy or contact dermatitis induced in particular by the latex, the plaster, the local anesthetic, the local antiseptic or one of the components of the study products
5. Subject with a recognized addiction to alcoholism or drug
6. Subject having already presented cicatrization disorders
7. Subject presenting pathology of cicatrization or pathology with consequences on cicatrization such as diabetes.
8. Subject with a disease including skin disease in progress on the upper limbs that may interfere with the interpretation of the evaluation criteria according to the opinion of the investigator
9. Subject with a history of atopic eczema on the upper limbs (arms, forearms, hands)
10. Subject with skin lesion, solar erythema, tanning marks, scar(s), tattoo(s), significant hair growth, nevus, visible or prominent veins on study areas that may interfere with the interpretation of the evaluation criteria according to the opinion of the investigator
11. Subject presenting hereditary disorders or acquired haemostasis
12. Subject with congenital methemoglobinemia or porphyria
13. Subject with chronic or acute pathology able to interfere with the results of the study according to the opinion of the investigator

Treatment and/or products:

14. Diuretic or diuretic systemic treatment initiated or modified in the 2 months prior to the inclusion visit or started at the inclusion visit or whose start is scheduled during the study
15. Treatment liable to induce methemoglobinemia (sulfonamides, dapsone, metoclopramide, flutamide, sodium nitroprusside) in the 14 days prior to the inclusion visit or during the inclusion visit
16. Systemic corticosteroid treatment in the 14 days prior to the inclusion visit or ongoing during the inclusion visit or planned during the study

17. Systemic treatment that may affect haemostasis, including anti-coagulant, antiplatelet therapy, within 7 days prior to the inclusion visit or ongoing at the inclusion visit or planned during the study
18. Systemic treatment with non-steroidal anti-inflammatory drug in the 7 days prior to the inclusion visit or ongoing at the inclusion visit (from 3 days of intake)
19. Systemic treatment liable to affect the healing process according to the opinion of the investigator in the weeks prior to the inclusion visit, ongoing at the inclusion visit or planned during the study
20. Systemic or topical antibiotic and/or antifungal treatment within 4 weeks prior to the inclusion visit or ongoing at the inclusion visit
21. Topical antiseptic treatment applied to the upper limbs (including the hands) within 2 weeks prior to the inclusion visit or ongoing at the inclusion visit.
22. Topical non-steroidal anti-inflammatory treatment, topical corticosteroids, immunomodulators applied to the upper limbs (including the hands) in the 15 days prior to the inclusion visit or ongoing at the inclusion visit or planned during the study
23. Physical treatment including forearm radiotherapy on the forearm in the 6 months prior to the inclusion visit or ongoing at the inclusion visit or planned during the study
24. Phototherapy treatment on the forearm in the 8 weeks prior to the inclusion visit, ongoing at the inclusion visit or planned during the study
25. Subject with intense and prolonged exposure to UV (natural or artificial) in the forearms in the 2 weeks prior to the inclusion visit or planned during the study
26. Any other treatments taken, applied or modified in the weeks prior to the inclusion visit, ongoing at the inclusion visit or planned during the study that may interfere with the study according to the opinion of the investigator
27. Use of keratolytic, exfoliating or self-tanning products on the upper limbs (including hands) in the 14 days prior to the inclusion visit or on the day of the inclusion visit
28. Use of a skin care product on the upper limbs (including the hands) in the 7 days prior to the inclusion visit or the day of the inclusion visit
29. Application of water, hygiene products on the upper limbs in the 8 hours prior to the inclusion visit
30. Subject having modified his usual body hygiene routine the day before the inclusion visit or planned to modify during the study.

Supplementary Figure 1. Time taken for re-epithelialization to occur in untreated (open symbols) and formula-treated (black symbols) wounded skin. For Subjects 1, 13 and 14, the re-epithelialization times were the same in untreated (open circles) and formula-treated (black circles) skin samples.

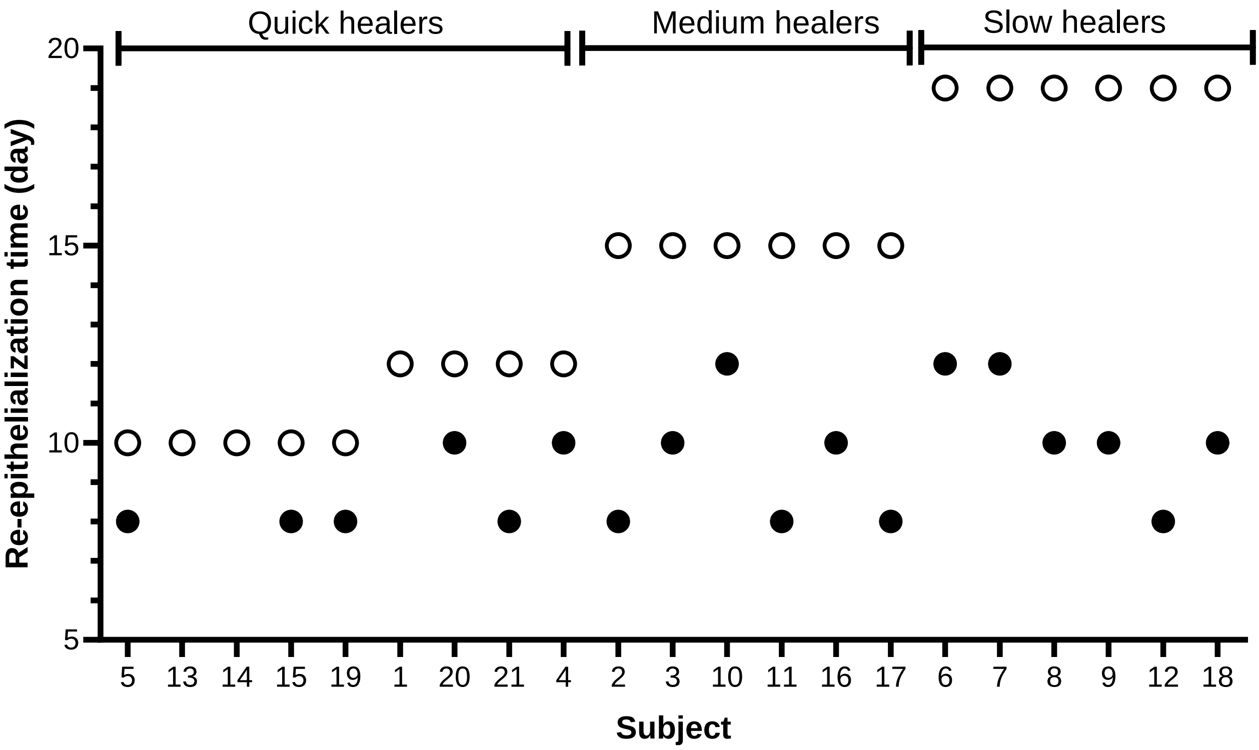

Supplementary Table 1. Forward and reverse primers and probes used for bacterial and fungal strains quantified using droplet digital PCR (ddPCR).

Analyzed Strains	Reference strains	Primers/probe	Amplicon size
Staphylococcus epidermidis	ATCC 12228D-5<br>PCI 1200	SodA gene<br>Forward 5’ TTTAGAAGCTAAATCAATCGAAGAAA 3’<br>Reverse 5’ GGTGACCACCGCCATTATTA 3’<br>Probe 5’ TGCCATCTAATATTCAAACAGCTGT 3’ (FAM)	95 bp
Cutibacterium acnes	ATCC 6919<br>NCTC 737	Lipase/acylhydrolase gene<br>Forward 5’ GTGTCGAGGTCGAAGTCGTT 3’<br>Reverse 5’ GTGAAGGCTGCTGTGCATAA 3’<br>Probe 5’ AGCCTTCGCGTTATTGGACTC 3’ (HEX)	149 pb
Malassezia resticta	ATCC MYA-4611D-5<br>CBS 7877	5.8S rRNA gene<br>Forward 5’ GGCGGCCAAGCAGTGTTT 3’<br>Reverse 5’ AACCAAACATTCCTCCTTTAGGTGA 3’<br>Probe 5’ TTCTCCTGGCATGGCAT 3’ (HEX)	89 pb
Malassezia globosa	ATCC MYA-4612D-5<br>CBS 7966	5.8S rRNA gene<br>Forward 5’ GGCCAAGCGCGCTCT 3’<br>Reverse 5’ CCACAACCAAATGCTCTCCTACAG 3’<br>Probe 5’ ATCATCAGGCATAGCATG 3’ (FAM)	75 pb
